# Blood Level Omega-3 Fatty Acids as Risk Determinant Molecular Biomarker for Prostate Cancer

**DOI:** 10.1155/2013/875615

**Published:** 2013-03-25

**Authors:** Mishell Kris Sorongon-Legaspi, Michael Chua, Maria Christina Sio, Marcelino Morales

**Affiliations:** ^1^Department of Preventive and Community Medicine, St. Luke's College of Medicine, Sta. Ignaciana Street, 1102 Quezon City, Philippines; ^2^Institute of Urology, St. Luke's Medical Center, 279 E. Rodriguez Boulevard, Cathedral Heights, 1102 Quezon City, Philippines; ^3^Department of Head, Ear, Neck and Throat, St. Luke's Medical Center, 279 E. Rodriguez Boulevard, Cathedral Heights, 1102 Quezon City, Philippines

## Abstract

Previous researches involving dietary methods have shown conflicting findings. Authors sought to assess the association of prostate cancer risk with blood levels of omega-3 polyunsaturated fatty acids (n-3 PUFA) through a meta-analysis of human epidemiological studies in available online databases (July, 2012). After critical appraisal by two independent reviewers, Newcastle-Ottawa Quality Assessment Scale (NOQAS) was used to grade the studies. Six case control and six nested case control studies were included. Results showed nonsignificant association of overall effect estimates with total or advanced prostate cancer or high-grade tumor. High blood level of alpha-linolenic acid (ALA) had nonsignificant positive association with total prostate cancer risk. High blood level of docosapentaenoic acid (DPA) had significant negative association with total prostate cancer risk. Specific n-3 PUFA in fish oil, eicosapentaenoic acid (EPA), and docosahexaenoic acid (DHA) had positive association with high-grade prostate tumor risk only after adjustment of interstudy variability. There is evidence that high blood level of DPA that is linked with reduced total prostate cancer risk and elevated blood levels of fish oils, EPA, and DHA is associated with high-grade prostate tumor, but careful interpretation is needed due to intricate details involved in prostate carcinogenesis and N-3 PUFA metabolism.

## 1. Introduction 

Prostate cancer in the recent decades has been shown to cause remarkable morbidity and mortality among males [1–3]. Although epidemiological research has identified several risk factors that can contribute to prostate cancer development, such as increasing age, family history, and ethnicity, particularly African American background, recent evidence has also suggested a role for chronic prostatic inflammation [4–6]. As such, the positive potential benefits of anti-inflammatory agents in risk reduction and prevention of prostate cancer have been sought by researchers [7, 8]. Specifically, dietary components such as omega-3 polyunsaturated fatty acids (n-3 PUFA) are of interest due to their established cardiovascular benefit, neuroprotectiveness, and anti-inflammatory effects [9–12]. These dietary n-3 PUFA, especially short-chain n-3 PUFA, are found mainly in nuts and vegetables, while long-chain n-3 PUFA are largely obtained from marine fish oil and to lesser extent from conversion of alpha-linolenic acid (ALA). ALA, which is considered an essential short-chain fatty acid because it cannot be synthesized by the human body, is an important source of long-chain n-3 PUFA such as docosahexaenoic acid (DHA), docosapentaenoic acid (DPA), and eicosapentaenoic acid (EPA) [10–13]. However, studies involving dietary intake of n-3 PUFA have yielded conflicting and nonconclusive results [14–25]. A recent meta-analysis, for instance, has attributed the inconclusive results to pooled diverse study design, presence of confounding variables, and presence of biases [25]. Thus, recall bias when using dietary questionnaires can significantly affect results. Dietary assessment techniques were also variable and may not accurately and precisely measure an individual's fatty acid intake due to under- or overreporting [26–28]. In search of a more precise and reliable method of estimating fatty acid consumption, authors have turned to measurement of fatty acid contents of blood, tissue, or erythrocyte membranes, since plasma phospholipids were noted to reflect current and long-term fatty acid consumption [29–32]. 

To address the above-mentioned problem, the authors of the present paper conducted an updated meta-analysis of human observational studies estimating the association of blood levels of n-3 PUFA and their derivatives (together and separately) with the risk of prostate cancer. As described previously [33], relevant literature was critically reviewed in order to provide the best evidence through quantitative analysis and systematic appraisal of study quality and homogeneity. 

## 2. Method 

### 2.1. Identification of the Literature

Electronic databases were searched using Firefox, Opera browser, and Windows explorer in order to identify medical literature about n-3 PUFA and prostate cancer with no restriction for language. Up to July 2012, the following electronic databases were searched: MEDLINE, UNBOUND MEDLINE, EMBASE, Science Direct, OVID, and ProQuest (Database of Dissertation and Thesis and Cochrane Library, including the Cochrane Database of Systematic Reviews). MEDLINE Medical Subject Heading (MeSH) terms used were “omega-3 fatty acids” and “prostate neoplasm.” For other databases, search keywords used were the following: “prostate,” “cancer,” “carcinoma,” “neoplasm,” “tumor,” “omega,” “fatty acids,” and “polyunsaturated.” References from studies that met our inclusion criteria and review articles or textbooks of related topics were searched for potentially relevant titles. External peer reviewers were asked to identify additional relevant studies that might not be included in the draft. We also inquired from industry/nutrition experts to provide any unpublished data. Non-English literature was translated to English before analysis. 

### 2.2. Inclusion and Exclusion Criteria

Studies were included in the meta-analysis if they met the following criteria: description of blood levels of n-3 PUFA, with or without derivatives, as exposure, diagnosis of prostate cancer with or without tumor grade (advanced, high-grade tumor) as outcome, prospective or retrospective case-control design with human study population, and studies that reported estimated effect size, that is, relative risk (RR), hazard ratio, or odds ratio (OR), with corresponding confidence intervals pertaining to comparison of high n-3 PUFA blood levels to the reference group (lowest blood level). In case control studies the primary effect estimate is OR. However, when the incidence of the outcome of interest is low, the OR can be taken as a good approximation to the RR, and these two parameters can be considered equivalent. Studies excluded were the following: those dealing with tissue n-3 PUFA levels since highly variable concentrations and diverse methods of determination can affect the results of the study; animal and in vitro studies because the results may not correlate well with in vivo human physiologic outcome; cross-sectional and ecologic studies since they were unable to provide informative effect estimates [34]; and review articles and letters to the editors because only collation of information and opinions were discussed. 

### 2.3. Selection of the Literature

Two of three physician reviewers, one of whom was specializing in urology, independently evaluated the citations and abstracts. The reviewers identified potential article titles on n-3 PUFA and prostate cancer. Articles that either reviewer identified were ordered, including abstracts and titles. The two physician reviewers then independently scored each article obtained, and if any unresolved disagreement arose, the senior physician (urologist) would settle the issue. In all stages, critical appraisal was performed independently by two reviewers. 

**Figure 1:**
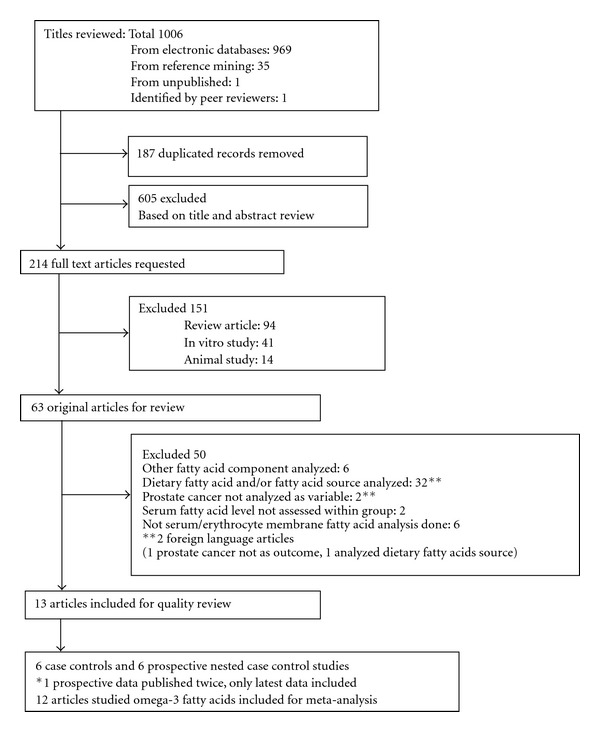
Prisma diagram of literature search and selection for meta-analysis.

A summary of the literature retrieval can be seen in Figure 1. A total of 1006 records were retrieved (969 from the electronic databases, 35 from manual reference mining, 1 unpublished from a graduate thesis, and 1 identified by external peer review). A total of 187 duplicated records were removed. A total of 605 records were excluded by the reviewers. In total, 214 articles were requested. On full text article review, 151 articles were excluded (in vitro, animal, and review studies). From the remaining 63 articles, 50 were excluded. Those excluded from meta-analysis were studies that investigated polyunsaturated fatty acids but did not specify n-3 PUFA or their components [35–40], those that determined fatty acids from dietary sources [41–72], those that did not consider prostate cancer diagnosis as an outcome [73, 74], or those that did not compare serum fatty acid levels within groups [75, 76], as well as studies that dealt with tissue fatty acids analysis [77–82]. Foreign language articles [83, 84] were included in the literature search, but none met the inclusion criteria for the meta-analysis. The unpublished article was not found to meet the inclusion criteria. 

### 2.4. Critical Appraisal of the Articles

The included articles were evaluated by the quality of the study design and its execution. By critical appraisal each study was scored according to the recommendation for review of epidemiological studies [85]. Each study design was evaluated based on the representative recruitment of the population, the baseline characteristics of the sample, measurement and ascertainment of cases and exposure, case and control selection/definition, description of withdrawals and dropouts, validity and reliability of the measurements (laboratory assessment of blood fatty acids), blinding of assessors, adjustment for confounders, extent of followup, calculation of effect size estimates given as OR or RR, size of confidence intervals (CI), Bradford Hills criteria, and applicability of the studies. A summary of each study's characteristics is seen in Table 1. Given that the maximal score was 11 points, a study that scored > 8 points would be included in the meta-analysis. If a study's quality score was rated below 8/11, then the two reviewers would discuss any discrepancies in their rating to derive a mutually accepted score and decide whether the study should be included. Afterwards, Newcastle-Ottawa Quality Assessment Scale (NOQAS) of Cochrane Collaboration on quality evaluation of descriptive studies for case control studies [86] was also used to grade all the articles included. NOQAS rating was used to further assess the quality of the studies and to aid in the statistical evaluation inasmuch as heterogeneity was noted between studies; it did not become the basis to weigh the individual effect estimates from each study. 

### 2.5. Data Extraction, Summary, and Statistical Analysis

One reviewer tabulated data from each study, and this was counterchecked by another reviewer. The reported RRs or ORs from each study were used to estimate the overall OR of prostate cancer patients showing the highest blood level of individual n-3 PUFA components (ALA, DHA, DPA, and EPA) versus the reference group. RR or OR and corresponding CI that had been adjusted to control for confounding variables were preferred whenever available in the publication. If a study's data had been published several times at different dates, only the most recent and comprehensive set was included. If an included study did not report any estimated effect measurement or sufficient raw data for the calculation of OR, the authors of the study were contacted by email with a request for the said data. The general variance-based method was used to analyze the prospective case control studies, because variance estimates were based on adjusted measures of effect and using 95% CI for the adjusted measure. CI was used because confounding variables are not ignored, and it is therefore superior in pooling observational data [87]. Each study's effect estimates (RR or OR) were converted to natural logarithms to stabilize the variances and expressed in risk ratios. The variance or standard error of the risk ratio was estimated from the CI. The overall odds ratio was estimated with the following: odds  ratio = exp⁡∑[*W*_*i*_ × ln⁡(OR_*i*_)]/∑*W*_*i*_, where *W*_*i*_ is a weight for the study, taken as the inverse of the variance. Heterogeneity was tested using Cochran's chi-square test (*Q*) to assess the consistency of associations, calculated by the following formula: *Q* = ∑[*W*_*i*_ × (ln⁡(ORs) − ln⁡(OR_*i*_))^2^] [87]. In cases of heterogeneity (*P* < 0.1), the source of the heterogeneity was identified by performing subgroup analyses on the basis of important differences in study design, that is, case control versus prospective studies. Nested case control studies are, like cohort studies, temporally prospective. Data from these studies were analyzed together, distinct from retrospective case control studies. Once the reasons for the observed heterogeneity were determined by subgroup analysis, the between-studies variance (*I* ^2^) was estimated in order to quantify the extent of heterogeneity among the pool. The *I* ^2^ statistic was used to describe the proportion of total variance in estimates of the RR due to heterogeneity. Sensitivity analysis was conducted by repeating the meta-analysis but excluding one study at a time from the pool of significantly heterogeneous designs (from the lowest NOQAS quality score to the highest) to assess the individual influence of each study on the overall effect estimate. Repeat meta-analysis was done until the least heterogeneity (*P* > 0.1) was noted in the sensitivity analysis. 

A random effect model was used to determine pooled effect estimates, since this model reflects a more conservative approach [88, 89]. For the purpose of analyzing the combined effect of long-chain n-3 PUFA (DPA + DHA + EPA) and commercially available fish oil n-3 PUFA (DHA + EPA) on the risk of prostate cancer and its subcategories, a mixed effect analysis-random effects model was used to combine data for each subgroup of long-chain n-3 PUFA. A fixed effect model was used to combine subgroups and yield the overall effect. The investigators used Comprehensive Meta-Analysis software version 2 by Biostat, Englewood, NJ [90], for statistical analysis of pooled data, and forest plots were constructed to illustrate pooled relative risks, wherein the point estimates for each effect were sized according to the inverse of the variance for each study. Publication bias was examined by using Egger's regression intercept [91], Begg-Mazumdar rank correlation [92] analysis, and a visual inspection of funnel plots of standard error intercept with RRs or ORs [93]. 

## 3. Results

### 3.1. Study Characteristics

A total of 12 studies, that is, 6 case-control studies [94–99] and 6 nested case control studies, were included [100–105]. The result of “The Physician's Health Study” was published in two separate publications [102, 106] with different times of followup. Only the most recent or complete data source was included in the analysis [102]. The study by Ukoli et al. reported two different high-risk populations, African American and Nigerian, in two different publications. In the earlier publication, only the Nigerian population was used, but in the latter article the authors compared data from the earlier article [96] with that obtained in the African American population [98]. Both articles were included in the meta-analysis, but only data from the African American population was taken from the latter article. Table 1 gives a description of each study's characteristics needed for appraisal: their total score, NOQAS score, and the variable adjustments performed. All studies included in this meta-analysis uniformly generated RR estimates of prostate cancer between the groups of the population with the highest blood level n-3 PUFA and the reference group (the one with the lowest blood level). The age of the study population ranged from 40 to 86 years both for cases and controls, and the age of the cases was matched to that of the controls. Overall case to control ratio was 1 : 1.27 in all the studies combined, with a total number of 4516 prostate cancer cases, who were matched with 5728 controls. Most studies used a diagnosis of prostate cancer as the case definition [94–104], and the only exception is the one that used tumor grade (high or low) as outcome [105]. Six studies made use of histopathology to ascertain cases of prostate cancer [94–98, 105], while six studies used hospital or histopathology records or cancer registries [99–104]. Four studies included advanced stage prostate cancer (extension through the capsule) [95, 102–104]. Five studies included high-grade tumor (Gleason score ≥7) in their analysis of outcome [97, 102–105]. In determining exposure for both cases and controls, five studies used erythrocyte membrane fatty acids [94, 95, 97, 99, 104], and seven studies used serum fatty acids [96, 98, 100–103, 105]. All studies provided a detailed description of their laboratory procedures, and all appear to be methodologically sound. Four studies also utilized a certain diet questionnaire for further assessment [97, 99, 103, 104]. Among the variables most commonly adjusted for were well-established risk factors for prostate cancer, which could be possible confounders: age [94–100, 102–105], body mass index [97, 101, 103–105], family history of prostate cancer [96–98, 104, 105], and race [94, 97, 104, 105]. Education [96, 98, 101, 103, 104] was considered for adjustment due to probable detection bias. The studies have applied most of the methodology standards and had quality assessment scores ranging from 8 to 10/11. Using the Newcastle-Ottawa Quality Assessment Scale (NOQAS) which had three categories to consider, selection, comparability, and exposure, all of the studies garnered a maximum score or one point below it in the comparability and exposure categories. Under the selection category, four studies had 2/4 score [101–104], while the rest maintained a perfect or almost perfect score [94–100, 105]. The main limitations found in the selected studies were the lack of representativeness of the study population. Selection of the cases and controls might not be as strict as it should be in case control studies, and registries used for ascertainment of cases may not be updated or 100% accurate. 

### 3.2. Association of Blood Level Omega-3 PUFA and Prostate Cancer Risk

**Figure 2:**
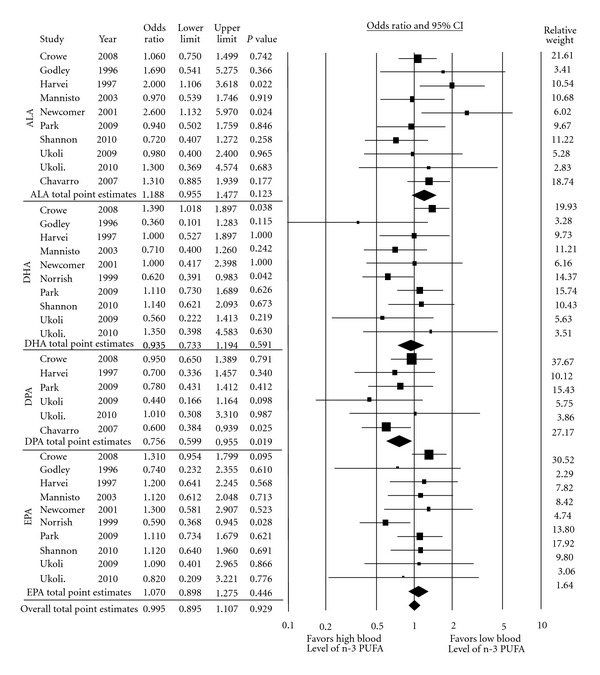
Forest plot of pooled effect estimate of blood level omega-3 PUFA on total prostate cancer risk.

**Figure 3:**
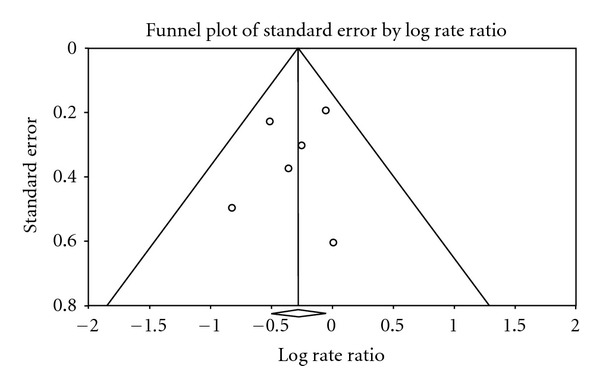
Publication bias determination using funnel plot.

**Figure 4:**
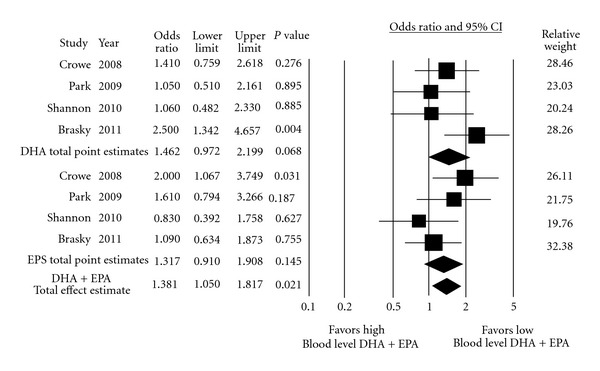
Forest plot of pooled effect estimate of blood level omega-3 PUFA on high-grade prostate tumor risk.

Pooled effect estimates with corresponding 95% CI from all included studies that described total prostate cancer occurrence, advanced prostate cancer, and high-grade tumor, respectively, and their association with blood level n-3 PUFA and different series/derivatives, ALA, DHA, DPA, and EPA (together and separately) are shown in Tables 2(a), 2(b), 3, and 4. These tables also describe the pooled sensitivity analysis, between study heterogeneity analyses and publication bias analysis using Begg's and Egger's methods. In particular, high blood levels of ALA were found to have a nonsignificant positive association to total prostate cancer risk (pooled OR: 1.188; CI: 0.955–1.477; *P* = 0.123) (Figure 2) with no significant heterogeneity (*P* = 0.240), although a small interstudy variation (*I* ^2^ = 22.065) was noted (Table 2(a)). Additionally, the pooled estimates of DPA had a significant association with total prostate cancer incidence (pooled OR: 0.756; CI: 0.599–0.955; *P* = 0.019) (Figure 2). Studies were noted to be homogeneous (*P* = 0.566) with no in-between study variation (*I* ^2^ = 0%). No publication bias was detected using either Begg's (*P* = 1.0) or Egger's (*P* = 0.54) approach (Table 2(a)), nor by visual inspection of the funnel plot (Figure 3). High blood levels of total n-3 PUFA or other series/derivatives (together and individually) were not found to have any significant association with total prostate cancer risk, advanced prostate cancer, or high-grade prostate tumor. In the analysis of blood level of DHA and EPA with total prostate cancer and high-grade prostate tumors, a significant heterogeneity was observed (Tables 2(b) and 4). The validity of pooling the above data may be uncertain because heterogeneity was observed and in-between study variation ranged from 32 to 53%. To identify the source of heterogeneity and interstudy variation, subgroup analysis was done using the variation in study design (nested case control versus case control) as seen in Table 2(b). After removal of the nested case control study that scored the lowest in NOQAS, namely, the Physician's Health Study [102], pooled estimate results showed reduced heterogeneity and decreased variation (*I* ^2^). After this adjustment was done, subgroup analyses showed no significant association between the individual long-chain n-3 PUFA series of DHA and EPA with total prostate cancer risk or its subcategories. Subgroup analysis was also done to determine the collective effect of long-chain n-3 PUFA (DPA + DHA + EPA) and fish oil content n-3 PUFA (DHA + EPA) on prostate cancer development. Fish oil n-3 PUFA was shown to have a positive association with high-grade prostate tumor risk (pooled OR: 1.381; CI: 1.050–1.817; *P* = 0.021) (Figure 4); adjusted interstudy heterogeneity was not significant (*P* = 0.291) with a small degree of interstudy variation (*I* ^2^ = 17.6%). Publication bias of the respective n-3 PUFA subgroup analyses was not evident using either Begg's (*P* = 0.734) or Egger's (*P* = 0.265, 0.952) tests (Table 4), or by visual inspection of the funnel plot (not shown). 

## 4. Discussion 

In this meta-analysis, results showed a positive association, though not significant, between high blood levels of ALA and prostate cancer risk. This finding does not coincide with the results of previous meta-analyses [14–25] which have suggested a protective effect of high dietary intake of this short-chain n-3 PUFA on prostate cancer development. A protective effect of dietary ALA supplementation might be linked to the bioconversion of ALA to EPA and DPA, which are potent anti-inflammatory mediators, producing a subclinical inflammation marker, matrix metalloproteinase-9 (MMP-9) that inhibits synthesis and release of cytokines [107–110]. In a meta-analysis of genomewide association studies, common minor alleles of single nucleotide polymorphisms (SNPs) in fatty acid desaturase-1 (FADS1) and -2 (FADS2) were found to be associated with higher serum levels of ALA and lower serum levels of EPA and DPA [111]. Possibly, high blood levels of ALA may suggest a genetic variation that no longer produces the positive effects brought about by ALA metabolism and bioconversion. Notably, there was a significant negative association between high levels of blood DPA level and prostate cancer risk with no significant heterogeneity, interstudy variability, or publication bias, which suggests that this long-chain n-3 PUFA may decrease the risk of prostate cancer development. DPA is abundant in whale meat, seal oil, and marine fatty fish, although in smaller quantities than EPA and DHA, in combination with which it is usually found [112]. In the human body, DPA can be synthesized mainly through bioconversion of EPA from ALA by the action of the chain elongating enzymes elongase-2 and elongase-5 and then retroconverted back to EPA in the liver and kidney [113–116]. Although few studies have been conducted regarding the physiological effects of DPA either in vitro or in vivo due to high production costs, studies have shown that DPA has potent activity in inhibiting the COX pathway of inflammation through enhanced formation of 12-hydroxy-5,8,10,14-eicosatetraenoic acid (12-HETE) in response to intact collagen or arachidonic acid, accelerating the lipoxygenase pathway (LOX), reducing platelet aggregation, and reducing age-related oxidative changes. It may have a potent inhibitory effect on angiogenesis through suppression of VEGFR-2 expression, and it may inhibit the expression of genes involved in inflammation, particularly TNF-induced necrotic cell death [117–121]. Some studies have, more specifically, identified the overexpression of proinflammatory COX-2 enzymes in prostate cancer cells to be a cause of cancer progression [122, 123]. The mentioned effects of DPA and mechanisms involved in inflammation and prostate carcinogenesis may suggest why high levels of DPA in the blood can reduce the risk of prostate cancer development. 

When the association of blood level DHA and EPA with prostate cancer and high-grade prostate cancer was determined, heterogeneity was noted. The source was found to be mostly the nested case cohort of the Physician's Health Study, possibly due to the selection of subjects and minimal adjustment of confounding variables. Compared to the general population, the research subjects were more conscious of health conditions affecting their diet and lifestyle, had more access to health care, were more compliant to followup, and generally were more knowledgeable. Thus, the information they have provided may have increased the validity of the result but also may have led to an early detection bias due to frequent followups and easier access to health care. Additionally, no adjustment was made for confounding variables that were known as risk factors for prostate cancer, such as family history, body mass index, and ethnicity. After adjusting the interstudy variability by removing the above-mentioned study from the pool, a significant positive association was noted between n-3 PUFA contained in fish oil (EPA + DHA) and high-grade prostate cancer, with no significant heterogeneity or publication bias from each subgroup (Table 4). Currently, studies still report inconsistent findings regarding the role of long-chain n-3 PUFA, such as EPA and DHA, in the development of prostate cancer. Some studies have identified EPA and DHA as ligands of peroxisome proliferator-activated receptors gamma (PPAR*γ*), nuclear factor kappa B, and retinoid X receptors, which have anti-inflammatory effects as well as antiproliferative effects in cancer cells [124]. EPA and DHA may also modulate the activity of cyclins and cyclin-dependent protein kinases in tumor cells, thus activating cancer cell apoptosis [125–128]. Other studies, in contrast, have suggested that long-chain n-3 PUFA can cause prostate carcinogenesis, and specifically the high grade or aggressive type [123, 128]. Recently, studies have mentioned that production of free radicals and reactive oxygen species occurs in the presence of long-chain n-3 PUFA, that is, DHA and EPA, in the prostate cells' beta-oxidative metabolic process. This leads to the formation of lipid hydroperoxides in the microenvironment of the cell that will further generate reactive species. The presence of these reactive oxygen species in the microenvironment promotes DNA mutation and, eventually, carcinogenesis [129]. Furthermore, prostate carcinogenesis also leads to augmentation of fatty acid oxidation, a major bioenergetic pathway, when dysplastic cells proliferate, to meet the energy requirement of rapid cell proliferation. Consequently, an increased fatty acid oxidation potentiates the progression of cancer into an aggressive type [130]. Environmental factors and sources of fish oil have also been reported to contribute to prostate cancer development. Two studies have reported that environmental toxins such as polychlorinated biphenyls or methylmercury compounds, which are found in contaminated marine fish, when consumed in the diet, can disrupt androgen and estrogen balance that may be associated with prostate cancer development [131, 132]. However, the involvement of these environmental toxins needs further research. 

Differences in genotypic components of COX-2, which modulates n-3 PUFA's effect on prostate cancer development, have also been investigated. Particular single nucleotide polymorphisms in the COX-2 gene were shown to alter the effects of long-chain n-3 PUFA in prostate cancer development. Two studies have identified that the variant alleles, COX-2 SNP rs5275 (+6364 A>G) and COX-2 SNP rs4648310 (+8897 A>G), found in men will maintain the inverse association between n-3 PUFA intake and prostate cancer, although more research is still needed to elucidate this issue [50, 55]. Lastly, however, these findings in genetic variation suggest that the effect of long-chain n-3 PUFA on prostate cancer development may vary among individuals depending on their differing genotype. 

### 4.1. Strength, Limitation, and Recommendations

Although observational studies have their intrinsic limits, they provide the only available data to explicate the relationship between n-3 PUFA and prostate cancer. The present meta-analysis employed a rigorous standard to assess methodological quality of the studies included in the analysis. Although heterogeneity was noted, the study design and sources of intervariability were assessed and adjusted to assure homogeneity of pooled data. Publication bias was not noted to be present among the included studies. The studies included in the present analysis were carried out in western countries, where diets are generally not healthy; thus the results extracted can only be generally applied to the western population. Additional studies with multiethnic or eastern populations are recommended to cover a more representative sample of the total population of human males. Though blood levels of n-3 PUFA reflect dietary intake, they can only provide insight in some aspects of the association between n-3 PUFA and prostate cancer, since the metabolism of n-3 PUFA is complex, and genetic variations may play a major role. More researches are recommended in order to elucidate the possible influence of genotypic variants, since few studies have been conducted. The contribution of environmental toxins to prostate cancer development also needs further research. 

## 5. Conclusion 

In conclusion, this meta-analysis provided evidence that elevated blood levels of DPA are associated with decreased risk of developing prostate cancer. Elevated blood levels of EPA and DHA in combination are associated with increased risk of high-grade prostate tumor. Cautious interpretation of these results must be done, since prostate carcinogenesis is multifactorial, and the body's metabolism of n-3 PUFA is complex. 

## Figures and Tables

**Table 1:** Summary of studies characteristics included in the meta-analysis.

								Quality score				
Study author year	Source	Study design	Age of study population (case/control)	Years of followup	Ascertain of cases (prostate ca)	Blood omega-3 fatty acid level determination	Level of comparison used	(NOQAS**)			Quality score (NHS^++^)	Adjustment variables
								S (4)	C (2)	E (3)		
Harvei 1997 [100]	Norway	Nested case control	Ave. 50 yo (141/282)	19.2 years (Ave. 11.6)	Cancer registries	Serum fatty acids	Quartiles	3	2	3	9/11	Age, area of residence

Männist$\ddot{\text{o}}$ 2003 [101]	Finland	Nested case control	50–69 yo (198/198)	5–10 years	Cancer registry and histopathology review	Serum fatty acids	Quartile	2	2	3	8/11	Age, area of residence (urban/rural), level of education, body mass index, alcohol consumption, and the number of years of smoking

Chavarro 2007 [102]	US	Nested case control	40–84 yo (476/476)	13 years	Hospital record and histopathology review	Blood level fatty acids	Quintile	2	1	3	9/11	Age, smoking status at baseline, and length of followup

Crowe 2008 [103]	Netherland	Nested case control	53–67 yo (962/1061)	4.2 years	National and regional cancer registry	Blood phospholipid	Quintile	2	2	3	9/11	Age, BMI, smoking, alcohol intake, level of education, marital status, and physical activity

Park 2009 [104]	USA	Nested case control	45–75 yo (376/729)	10 years	Tumor registry	Erythrocyte membrane fatty acids	Quartile and tertile	2	2	3	10/11	Age, area of residence, race/ethnicity, family history of prostate cancer, BMI, level of education, hour of fasting, date, and time of blood draws

Brasky 2011 [105]	US	Nested case control (from PCPT)	55–84 yo (1658/1803)	7 years	End-study prostate biopsies	Serum fatty acids	Quartile	4	2	3	9/11	Age, race, family history of prostate cancer, diabetes, BMI, alcohol, and treatment arm

Norrish 1999 [99]	New Zealand	Case control	40–80 yo (317/480)	N/A	Histopathology	Erythrocyte membrane fatty acids	Quartile	3	2	3	9/11	Age, height, total nonsteroidal anti-inflammatory drug use, socioeconomic status, and food frequency questionnaire-estimated intake of total polyunsaturated fat

Shannon 2010 [97]	US	Case control	50–86 yo (127/183)	N/A	Histopathology	Erythrocyte membrane fatty acids	Tertile	3	2	3	9/11	Age, BMI, race, and family history of prostate cancer

Godley 1996 [94]	US	Case control	>45 yo (89)(38)	N/A	Histopathology	Erythrocyte membrane fatty acids	Quartile	3	2	2	8/11	Age and Race

Newcomer 2001 [95]	US	Case control	41–66 yo (67/156)	N/A	Histopathology	Erythrocyte membrane fatty acids	Quartile	3	1	3	8/11	Age

Ukoli 2009 [96]	Nigeria	Case control	≥45 (66/226)	N/A	Histopathology	Serum fatty acids	Quartile	3	2	3	9/11	Age, level of education, family history of prostate cancer, and waist-hip ratio

Ukoli 2010 [98]	Nigeria	Case control	≥45 (48/96)	N/A	Histopathology	Serum fatty acids	Quartile	3	2	3	9/11	Age, level of education, family history of prostate cancer, and waist-hip ratio

**Newcastle-Ottawa Quality Assessment Score.

^++^National Health Service, UK, recommended critical appraisal of case control.

S: selection; C: comparability; E: exposure.

**Table:** (a)

Groups		Heterogeneity^∧^				Effect estimates and 95% confidence interval				Publication bias	
Omega-3 derivatives	Number of study	*Q* value	df	*P* value	*I* ^2^	Point estimates	Lower limit	Upper limit	*P* value	Begg	Egger
ALA	10	11.548	9	0.240	22.065	1.188	0.955	1.477	0.123	0.283	0.502
DHA	11	18.991	10	0.040	47.343	0.876	0.685	1.119	0.290	0.436	0.239
DHA^‡^	10	14.450	9	0.107	37.716	0.935	0.733	1.194	0.591	0.211	0.127
DPA	6	3.883	5	0.566	0.000	0.756	0.599	0.955	0.019	1.000	0.540
EPA	11	14.741	10	0.142	32.162	0.971	0.784	1.204	0.792	0.533	0.671
EPA^‡^	10	8.656	9	0.470	0.000	1.070	0.898	1.275	0.446	0.211	0.502
(DHA + DPA + EPA)*		32.676	25	0.139	23.492	0.942	0.834	1.064	0.336		
(DHA + EPA)*		23.410	19	0.220	18.840	1.022	0.887	1.179	0.760		
Total omega-3*		47.526	35	0.077	26.356	0.995	0.895	1.107	0.929		

**Table:** (b)

Groups		Heterogeneity^∧^				Effect estimates and 95% confidence interval				Publication bias	
Omega-3 derivatives	Number of study	*Q* value	df	*P* value	*I* ^2^	Point estimates	Lower limit	Upper limit	*P* value	Begg	Egger
ALA	10	11.548	9	0.240	22.065	1.188	0.955	1.477	0.123	0.283	0.502
Case control	5	6.893	4	0.142	41.971	1.237	0.735	2.083	0.423		
Nested case control	5	4.614	4	0.329	13.300	1.191	0.948	1.496	0.132		
DHA	11	18.991	10	0.040	47.343	0.876	0.685	1.119	0.290	0.436	0.239
Case control	6	5.433	5	0.365	7.972	0.769	0.558	1.060	0.109		
Nested case control	5	11.213	4	0.024	64.327	0.942	0.670	1.325	0.733		
DPA	6	3.883	5	0.566	0.000	0.756	0.599	0.955	0.019	1.000	0.540
Case control	2	1.126	1	0.289	11.180	0.620	0.278	1.382	0.243		
Nested case control	4	2.433	3	0.488	0.000	0.773	0.605	0.988	0.040		
EPA	11	14.741	10	0.142	32.162	0.971	0.784	1.204	0.792	0.533	0.671
Case control	6	4.603	5	0.466	0.000	0.851	0.634	1.143	0.285		
Nested case control	5	8.661	4	0.070	53.818	1.028	0.757	1.396	0.859		

^∧^Interstudy heterogeneity was tested by Cochrane's *Q* (Chi^2^) at a significance level of *P* < 0.10 and quantified by *I* ^2^, where *I* ^2^ ≥ 50% is considered to be evidence of substantial heterogeneity and ≥75%, considerable heterogeneity.

^‡^Interstudy variation adjusted (heterogeneous study removed from the pool of effect estimates).

*Generated from adjusted total effect estimates from each n-3 PUFA random effect analysis.

**Table 3:** Blood level omega-3 Polyunsaturated fatty acids versus advanced prostate risk random effect analysis model.

Groups		Heterogeneity				Effect estimates and 95% confidence interval				Publication bias	
Omega-3 derivatives	Number of study	*Q* value	df	*P* value	*I* ^2^	Point estimates	Lower limit	Upper limit	*P* value	Begg	Egger
ALA	3	0.654	2	0.721	0.000	0.965	0.576	1.618	0.893	0.296	0.051
DHA	4	2.289	3	0.515	0.000	0.896	0.640	1.256	0.524	1.000	0.342
DPA	3	0.367	2	0.832	0.000	0.870	0.514	1.473	0.606	1.000	0.618
EPA	4	6.180	3	0.103	41.457	0.975	0.582	1.634	0.925	0.308	0.309
(DHA + DPA + EPA)*		8.870	10	0.545	0.000	0.908	0.708	1.164	0.447		
(DHA + EPA)*		8.482	7	0.292	17.471	0.919	0.693	1.219	0.559		
Total omega-3*		9.580	13	0.728	0.000	0.919	0.734	1.149	0.457		

*Generated from total effect estimates from each n-3 PUFA random effect analysis.

**Table 4:** Blood level omega-3 polyunsaturated fatty acids versus high-grade prostate risk random effect analysis model.

Groups		Heterogeneity^∧^				Effect estimates and 95% confidence interval				Publication bias	
Omega-3 derivatives	Number of study	*Q* value	df	*P* value	*I* ^2^	Point estimates	Lower limit	Upper limit	*P* value	Begg	Egger
ALA	5	7.731	4	0.102	48.264	0.965	0.605	1.538	0.881	0.807	0.870
DHA	5	8.593	4	0.072	53.449	1.233	0.769	1.978	0.385	0.221	0.051
DHA^‡^	4	4.310	3	0.230	30.389	1.462	0.972	2.199	0.068	0.734	0.265
DPA	3	3.291	2	0.193	39.231	0.597	0.299	1.193	0.144	1.000	0.930
EPA	5	8.362	4	0.079	52.162	1.130	0.717	1.781	0.599	0.221	0.273
EPA^‡^	4	3.931	3	0.269	23.675	1.317	0.910	1.908	0.145	0.734	0.952
(DHA + DPA + EPA)*		20.370	10	0.026	50.908	1.232	0.955	1.590	0.108		
(DHA + EPA)*		8.498	7	0.291	17.629	1.381	1.050	1.817	0.021		
Total omega-3*		29.708	15	0.013	49.508	1.165	0.931	1.457	0.181		

^∧^Interstudy heterogeneity was tested by Cochrane's *Q* (Chi^2^) at a significance level of *P* < 0.10 and quantified by *I* ^2^, where *I* ^2^ ≥ 50% is considered to be evidence of substantial heterogeneity and ≥75%, considerable heterogeneity.

^‡^Interstudy variation adjusted (heterogeneous study removed from the pool of effect estimates).

*Generated from adjusted total effect estimates from each n-3 PUFA random effect analysis.
